# 

**Published:** 1900-05-19

**Authors:** 


### NOTICE TO CORRESPONDENTS.

All MS., Letters, Books for Review, and other matters intended for the Editor should be addressed The Editor, "The Hospital," The Hospital Building, 28 & 29, Southampton Street, Strand, W.C.

The Editor cannot undertake to return rejected MS., even when accompanied by stamped directed envelope.

**Notice to Advertisers and Subscribers.**

All Advertisements, Orders for Copies of The Hospital, and Business Communications should be addressed to **THE MANAGER (Not to the Editor),** The Hospital, The Hospital Building, 28 & 29, Southampton Street, Strand, London, W.C.

The Cost of Subscription, post free, is as follows:—Per week, 2½d.; per quarter, 2s. 8d.; per half-year, 5s. 3d.; per annum, 10s. 6d. Foreign:—Per annum, 15s. 2d., payable, in all cases, in advance.

**NOTICE.—Vol. XXVII. of "The Hospital" (October, 1899, to April, 1800), in handsome cloth binding, is now on sale at the Office of this Journal, price 7s. 6d. Cases for binding the Numbers contained in this Volume 1s. 6d. Index to Vol. XXVII., 2½d., post free.**

**The Monthly Part for June will be ready on June 1. Price 6d., by post 8d.**

### SCRAPS AND GLEANINGS.

THE new Royal Victoria Hospital, shortly to be erected in Belfast in commemoration of the Diamond Jubilee, will cost over £100,000.

THE total number of in-patients admitted during the year to St. Bartholomew's Hospital, Chatham, was 1,035. The out-patients numbered during the year 15,429.

DURING the past year the number of in-patient cases treated at the North Riding Infirmary was 896, as against 800 the previous year. The out-patients numbered 3,461.

OWING to a technical and legal point, the Building Committee of the Folkestone Town Council are to reconsider their recommendations in reference to the plans of the additions to the Victoria Hospital.

THE Salford Town Council have applied to the Local Government Board for permission to borrow £3,000 for the purchase of a site and the necessary building for the purpose of the provision of a small-pox hospital.

AT the recent annual meeting of the Nottingham Hospital Saturday Committee it was announced that the total amount of the Hospital Saturday Fund collections up to date was £3,250, being an increase of £240 upon last year's amount.

AN exhibition of children's portraits by Mr. R. N. Speaight was opened free to the public at 178, Regent Street on May 11th, and will remain open till August 4th. The exhibition includes photographs of a number of children of the Royal Family.

THE appeal for £500 to provide 10 beds for the Scottish Redcross Hospital has met with a liberal response, and £531 odd has already been received. A sum of £10 odd is now being asked for to make up the amount necessary to equip the eleventh bed.

THE total number of patients under treatment at the Leicester Infirmary during 1899 was far in excess of any other year in the institution's history. The number of out-patients treated during 1899 (viz., 17,451) was almost exactly double the number treated in 1879.

THE new fever hospital for Hawick, about to be erected by the Hawick Corporation and the Hawick District Committee of the County Council, is estimated to cost a total sum of £6,000, £1,000 of which is payable by the Hawick District Committee, and the remainder by the burgh of Hawick.

THE buildings for the new burgh hospital for infectious diseases at North Berwick have cost over £2,560. Although the building is designed for two ward blocks, only one, containing two wards of four beds each, has at present been erected, together with the administration block and ward offices.

ON his retirement, after upwards of 10 years' service, from the chairmanship of the board of management of the Royal Albert Edward Infirmary, Wigan, Mr. James Brown was presented by the friends of the institution with his portrait painted in oils, and with a silver tea and coffee service for Mrs. Brown.

### THE STUDENT OF MEDICINE.

### APPOINTMENTS.

S. BOAKE, L.R.C.P., L.R.C.S.I., appointed Medical Officer for the Ilminster No. 1 District of the Chard Union. E. T. Collins, F.R.C.S.Eng., appointed Surgeon to the Royal London Ophthalmic Hospital. J. H. Fisher, M.B., B.S.Lond., F.R.C.S.Eng., appointed Assistant Surgeon to the Royal London Ophthalmic Hospital. P. Flemming, M.D.Lond., F.R.C.S.Eng., appointed Assistant Surgeon to the Royal London Ophthalmic Hospital. C. Gordon, M.B.Edin., appointed Medical Referee under the Workmen's Compensation Act, 1897, to County Court Circuit No. 57. G. O. Jacobsen, L.R.C.P.Lond., M.R.C.S.Eng., appointed Medical Officer for the Fourth District of the Chipping Norton Union. R. Kennedy, M.A., M.D., D.Sc.Glasg., appointed Assistant Surgeon to the Western Infirmary, Glasgow. A. Lawson, F.R.C.S.Eng., appointed Assistant Surgeon to the Royal London Ophthalmic Hospital. E. R. A. MacDonnell, L.R.C.P., L.R.C.S.Edin., appointed Medical Officer for the Litcham District of the Mitford and Launditch Union. H. J. M'Nabb, M.B.R.U.I., appointed Assistant Medical Officer to the co. Down Infirmary. G. Newman, M.D.Edin., D.Ph.Camb., appointed Medical Officer of Health for Clerkenwell. A. S. Norman, L.R.C.P., M.R.C.S.-Eng., D.P.H.Camb., appointed Medical Officer for the Second District and Workhouse of the Havant Union. G. E. Rennie, M.D., M.R.C.P.Lond., appointed Honorary Assistant Physician to Prince Alfred Hospital, Sydney, and Acting Tutor in Clinical Medicine, University of Sydney. J. F. Rimmer, M.B., Ch.B.-Vict., appointed Resident Assistant Medical Officer to the Workhouse of the Birkenhead Union. K. Roberts, M.R.C.S.Eng., L.R.C.P.Lond., appointed Medical Officer of Health for the combined Ampthill and Woburn Rural Districts. W. T. H. Spicer, M.A., M.B.Camb., F.R.C.S.Eng., appointed Surgeon to the Royal London Ophthalmic Hospital.

NATIONAL HOSPITAL FOR THE PARALYSED AND EPILEPTIC, QUEEN SQUARE.—The following staff promotions and appointments have been recently made: Consulting Physicians (retaining charge of a certain number of beds): J. H. Jackson, M.D., F.R.C.P.Lond., F.R.S.; T. Buzzard, M.D., F.R.C.P.Lond. Physicians: D. Ferrier, M.D., F.R.C.P.Lond., F.R.S.; J. A. Ormerod, M.D., F.R.C.P.Lond. Physicians for Out-patients: J. Taylor, M.D., F.R.C.P.Lond.; J. R. Russell, M.D., F.R.C.P.-Lond. Assistant Physicians: W. A. Turner, M.D., F.R.C.P.-Lond.; F. E. Batten, M.D., M.R.C.P.Lond.

28 & 29, SOUTHAMPTON STREET, STRAND, LONDON, W.C.

